# Endophytes and their potential in biotic stress management and crop production

**DOI:** 10.3389/fmicb.2022.933017

**Published:** 2022-10-17

**Authors:** Parul Chaudhary, Upasana Agri, Anuj Chaudhary, Ashish Kumar, Govind Kumar

**Affiliations:** ^1^Govind Ballabh Pant University of Agriculture and Technology, Pantnagar, Uttarakhand, India; ^2^Shobhit University, Gangoh, Uttar Pradesh, India; ^3^Indian Council of Agricultural Research (ICAR)-Central Institute for Subtropical Horticulture, Lucknow, India

**Keywords:** biotic stress, endophytes, plant growth, sustainable agriculture, biocontrol

## Abstract

Biotic stress is caused by harmful microbes that prevent plants from growing normally and also having numerous negative effects on agriculture crops globally. Many biotic factors such as bacteria, fungi, virus, weeds, insects, and nematodes are the major constrains of stress that tends to increase the reactive oxygen species that affect the physiological and molecular functioning of plants and also led to the decrease in crop productivity. Bacterial and fungal endophytes are the solution to overcome the tasks faced with conventional farming, and these are environment friendly microbial commodities that colonize in plant tissues without causing any damage. Endophytes play an important role in host fitness, uptake of nutrients, synthesis of phytohormone and diminish the injury triggered by pathogens *via* antibiosis, production of lytic enzymes, secondary metabolites, and hormone activation. They are also reported to help plants in coping with biotic stress, improving crops and soil health, respectively. Therefore, usage of endophytes as biofertilizers and biocontrol agent have developed an eco-friendly substitute to destructive chemicals for plant development and also in mitigation of biotic stress. Thus, this review highlighted the potential role of endophytes as biofertilizers, biocontrol agent, and in mitigation of biotic stress for maintenance of plant development and soil health for sustainable agriculture.

## Introduction

Agricultural strengthening is an important factor to the food safety for the rising world population. The recovery of soil fertility and crop heath by the usage of chemical fertilizers not only affects the soil health by decreasing the water holding capacity, depleting soil fertility, and diminishing soil nutrient and microflora but also poses a threat to human health and ecosystem. By considering all these problems, researchers are attentive for the substitution of chemical fertilizers with microbial-based fertilizers (Granada et al., 2018). Application of endophytes as biofertilizers can be a better approach to improve soil microbial status that stimulates the natural soil microbiota, therefore influencing nutrient accessibility and decomposition of organic matter (Fasusi et al., 2021). Endophytes are microbes that live within the host plant and have the capability to colonize plant roots without causing harm to the plants. They increase plant growth, act as biocontrol agent and protect the host from pest naturally, and endure tolerance against numerous biotic/abiotic stresses. Endophytes capable of producing several growth hormones such as IAA, ACC deaminase, increased in uptake of K ions in plant tissues, and decreased ethylene level are an alternate mechanism to alleviate stress conditions in various plants (Fan et al., 2020; Agri et al., 2022). They are also able to improve the uptake of nutrients such as nitrogen, magnesium, zinc, sulfur, and phosphorus from soil and provide to the host plant for better growth and survival (Agri et al., 2021).

Both bacterial and fungal endophytes hold tremendous potential for being used as biocontrol agent. Endophytes show antagonistic activity against disease-causing phytopathogens and diminish the damage attributed to phytopathogens. They produce several bioactive antimicrobial and antiviral metabolites along with producing various antioxidants to suppress pathogens (Gouda et al., 2016). Moreover, diverse range of fungal species especially entomopathogenic fungi have been known to exert long-term preventive measure for insect population (Litwin et al., 2020). Different bacteria such as *Bacillus*, *Pseudomonas*, *Pedobacter*, and *Acidobacterium* involved in mineral solubilization, metabolite production, and N_2_ fixation. Several fungal strains including *Beauveria bassiana*, *B. metarhizium*, *M. robertsii*, *Chaetomium globosum*, and *Acremonium* spp. are successful in plant protection (Grabka et al., 2022). With a wide host range, endophytic fungus becomes advantageous as compared to other biocontrol agents. Notably, *Trichoderma viride* isolated from *Spilanthes paniculata* showed broad range activity against *Colletotrichum capsici*, *Fusarium solani*, and *Pythium aphanidermatum* (Qi et al., 2019).

Crop plants undergo various environmental stresses during their growth period that ultimately results in reduced crop productivity. Genetic and physical growth alteration due to several environmental cues restricts the full plant development in their growth period. One such biotic stress occurs by the recurrent attack on plants by phytopathogens such as bacteria, virus, fungi, and herbivores, which ultimately reduce plant vigor and death of host plant in extreme conditions (Pandey et al., 2017). In agricultural field, biotic stress especially caused by bacteria and fungal phytopathogens is the major cause of pre- and post-harvest losses. Plant being sessile in nature responds to stress conditions accordingly through various stimulatory mechanisms. They have evolved unique physiological, biological, and molecular adaptation strategies to adjust the adverse conditions and promote plant growth. However, the extent of stress and climatic extremity makes them unable to cope up with the challenges raised by the environment (Chitnis et al., 2020). The generalized defense system in plants is unable to fully relieve the pressure and meet the demands of multistress tolerance to thrive and survive. So far, genetic engineering and other chemical and physical methods have been used to get stress tolerant cultivars. But they do not provide stress tolerance capacity for a very long time, and also, they are not ecofriendly. Thus, harnessing the potential of beneficial endophytes present in the nature for disease management could be an alternative strategy for improving plant resistance and resilience in crop varieties (Zheng et al., 2021). This will not only reduce chemical inputs but mitigate environmental stress without causing adverse effects. Useful endophytic microbes residing in the plant tissues are promising measure to remediate stressful conditions in a natural way.

## Endophytes

Plants are associated with a wide range of microbial community having positive, negative, or neutral kind of response in their host plant. Majority of the research is focused on the known epiphytic beneficial microbes colonizing the rhizosphere zones. However, plant growth-promoting endophytes are the subset of rhizosphere microbiome that is important determinants of plant microecosystems (Khati et al., 2018; Chaudhary et al., 2021a). The potential of endophytes as a bioinoculant is thus far to be sightseen to the completest potential due to few shortcomings. Such endosymbiont groups of microbes are diverse and harbored in almost every other plant species found in nature (Nair and Padmavathy, 2014). They mutually reside and proliferate within the plant tissues such as stems, roots, seeds, fruit, buds, and leaves deprived of producing any damage to the host plants (Specian et al., 2012). A small change in the diversity of plant endophytic communities can have significant impact over plant growth regulation and environmental adaptation (Vandenkoornhuyse et al., 2015). Gradual co-evolution in plant endophytic associations has eventually led to a positive response toward each other existence and influence vital activities in their host plant (Wang and Dai, 2011).

Endophytes are potent microbial resource needed to be explored for their application in agriculture sector. Most of the beneficial growth-promoting species belongs to the facultative group of endophytes that live in soil freely but colonizes crop plants under suitable conditions (Gaiero et al., 2013). Almost every other plant species hosts various bacterial, fungal, or actinomycete endophytes that may regulate plant and soil health. Various plant growth parameters are regulated by the colonization of endophytes and based on the microenvironment and the host’s metabolic capacity; they biosynthesize various compounds emanating growth-promoting activities similar to rhizospheric microbes (Chaudhary et al., 2021b,^2022^). They maintain stable symbiosis through secreting various bioactive compounds contributing to colonization and plant growth (Gouda et al., 2016). The attributes associated with endophytes include the production of extracellular enzymes (Khan et al., 2014), bioremediation, synthesis of secondary metabolites against phytopathogens (Mousa and Raizada, 2013), and induced systemic resistance (Constantin et al., 2019). But mainly, endophytic bacterial and fungal strains confer propound impacts on the overall health and maintenance of crop plants under different environmental conditions *via* nitrogen fixation, phosphate solubilization, siderophore, and phytohormones production and by conferring tolerance to various stresses. Additionally, N-fixing endophytes *Novosphingobium sediminicola*, *Ochrobactrum intermedium* (from sugarcane) and *Bradyrhizobium*, *Kosakonia*, and *Paraburkholderia* (from rice) carry nitrogen fixation genes (Muangthong et al., 2015; Okamoto et al., 2021).

Both climatic and edaphic factors equally contribute to the nature and action of endophytes toward plants (Kandel et al., 2017a). Under different condition, they also enhance the levels of plant growth-promoting hormones (cytokines, gibberellins, and auxin) and facilitate nutrient cycling whenever required (Egamberdieva et al., 2017; Chaudhary and Sharma, 2019). Few are known to produce polyamines, including putrescine, cadaverine, spermidine, and spermine, which involved in lateral root development and stress adaptations (Couee et al., 2004). Numerous growth-promoting bacterial and fungal endophytes have been reported till date. Microbial symbionts are suitable to maximize crop productivity, but more research is required to understand the significance in plant growth (Chaudhary et al., 2021c). However, complete understanding of the mechanisms and the genetic regulation utilized by endophytes in plant growth regulation is an important aspect to be studied for their application under field conditions.

## Diversity and distribution of endophytic microbes for maintenance of soil health and plant productivity

### Microbial root endophytes

Roots are the main habitat and colonization route for the bacterial and fungal endophytes. The main entry points for bacterial colonization are root hairs, root cracks, or wounds formed by microbial or nematode activities. The other major sites for root colonization include intercellular spaces in cortex and epidermis (Compant et al., 2005). Endophytes such as *Pseudomonas putida* and *P. fluorescens* colonized the olive through root hairs (Mercado-Blanco and Prieto, 2012). An axenically phytopromotional fungal root endophyte *Piriformospora indica* begins root colonization in the cortex region by a biotropic growth phase and continues with cell death-dependent phase. Inoculation of *P. indica* promotes plant growth, early flowering, higher seed yield, and adaptation to stresses in various host plants such as *Phaseolus vulgaris*, *Triticum aestivum*, and *Cicer arientum* (Varma et al., 2012; Ansari et al., 2014).

Rhizospheric microorganisms are enriched with nutrients and influence plant growth through soil nutrient recycling and nutrient uptake (Kukreti et al., 2020; Kumari et al., 2020). Overall root endosphere is metagenomically diverse and most often dominated by beneficial *Proteobacteria* (50%), *Actinobacteria*, *Firmicutes*, and *Bacteriodetes* (10%) (Liu et al., 2017). In association with roots, such microbe produces several compounds that influence plant development. Plant hormones such as gibberellins, cytokinins, and indole acetic acid (IAA) highly facilitate plant growth. In addition, few are known to promote plant mycorrhization. For instance, ACC deaminase (1-amonocyclopropane-1-carboxylic acid) containing *Arthrobacter protophorniae* enhanced nodulation in *Pisum sativum* (Barnawal et al., 2014). The other best-known fungal root colonizers are known as dark septate endophytes (DSE). The *Phialocephala fortinii* s.l- Acephala applanata species complex (PAC) species of *Ascomycetes* are the DSE fungi in forestry systems. In the study, dual inoculation of PAC positively increases plant biomass in spruce (Reininger and Sieber, 2013).

Endophytes living under extreme conditions such as Antarctica are also known to boost crop productivity. Under stressed condition, where mycorrhizae are generally low in abundance, different fungal endophytes potentially act as the prime root mutualistic symbionts (Mandyam et al., 2010). In terms of increasing nutrient acquisition of nutrients such as phosphorus from the roots and increasing the host fitness, both root-associated endophytes and mycorrhizal fungi provide benefits in a very similar manner. However, furthermost fungal endophytes do not endure an obligate biotrophic life phase and live at smallest part of their life cycle separated from the plant (Park and Eom, 2007). The only two known vascular plant, i.e., *Colobanthus quitensis* and *Deschampsia Antarctica* from such extreme condition harbors *Penicillium* species. *Penicillium* (root endophyte) helps in growth of vascular plants in Antarctic region *via* enhancing nitrogen acquisition and nutrient uptake by significantly increasing yield. The mechanism involved in nitrogen acquisition is attributed to the litter protein breakdown and amino acid mineralization (Oses-Pedraza et al., 2020). In total, two fungal strains isolated from Antarctic plants rhizosphere, i.e., *Penicillium brevicompactum* and *P. chrysogenum* isolated from plants rhizosphere, i.e., *Colobanthus quitensis* and *Deschampsia Antarctica* increased the final yield by 42% in lettuce and 68% in tomato plants in comparison with control (Molina-Montenegro et al., 2020). Several genera of beneficial root endophytes have been reported from medicinal plants such as *Pseudomonas*, *Xanthomonas*, *Bacillus*, *Inquilinus*, and *Pedobacter.* They have been associated with stimulation of growth activities such as production of secondary metabolites, solubilizing phosphate, and upregulating the expression of certain stress regulating genes under stress conditions (Rat et al., 2021).

Horizontal transmission colonization of the root endosphere *via* the rhizosphere. Types of endophytes: Passive endophytes – They penetrate through cracks present at root emergence area, root tips, or those created by pathogens; facultative endophytes – They live exterior to the host in certain phase of their life cycle and are frequently allied with plants from its adjoining soil; obligate endophytes – they depend plant metabolism for their survival; endofungal bacteria – Bacterial symbionts of fungi occur inside fungal spores and hyphae.

## Endophytic community in aerial tissues (phyllosphere)

Not all endophytes enter *via* root zones and move through the xylem vessels, they harbor diverse communities that enter the aerial tissues *via* above-ground surfaces too. Different entry routes chosen by many plant-promoting endophytes are stem (laimosphere), fruits (carposphere), leaves (Phyllosphere), seeds (spermasphere), and flowers (anthosphere) (Lindow and Brandl, 2003). Endophytes that live within the leaf tissues and stems are well documented. Phyllosphere microbes are an important component of microbial communities that live asymptomatically within leaves and also known for plant health maintenance (Ritpitakphong et al., 2016). Besides being the largest microbial habitat on Earth, the functional roles of phyllosphere residents are still less understood over the rhizosphere microbiome. It is estimated that their abundance in nature may exceed 10^62^ cells globally. *Proteobacteria*, *Actinobacteria*, and *Bacteroidetes* were the most abundant phyla associated with *A. thaliana*, *Populas*, and *Salix* (Redford et al., 2010; Firrincieli et al., 2020). The most abundant genus of phyllosphere region is *Pseudomonas* in tomato plants (Dong et al., 2019). Leaf endophytes including bacteria and fungi are the subset of phyllosphere endophytes. Leaf endophytes most of the times comprise five phyla, *Proteobacteria* (90%), *Actinobacteria* (2.5%), *Plancomycetes* (1.4%), *Verrucomicrobia*, and *Acidobacteria* (1.1 and 0.5%) (Romero et al., 2014). They live inside the leaf and maintain symbiotic relationship with the host plants.

It is evident to suggest that endophytes enter leaves and stems through openings such as stomata and hydathodes through dispersion with the help of rain, soil, or pollinators (Frank et al., 2017). For instance, *Gluconobacter diazotrophicus* enters through stomata in sugarcane plants (James et al., 2001). After reaching this site, endophyte strains multiply and form a thin layer of biofilm. Apart from this, some may enter to the inner tissues and start residing as endophytes where further microbes could colonize themselves into xylem. They further colonize and multiply in different organs including anthrosphere, phylloplane, carposphere, and caulosphere (Meyer and Leveau, 2012). Numerous growth-promoting foliar endophytes have been identified through high-throughput screening procedures. Despite this, the gaps still hinder their field application and practical exploitation in agriculture. Not only bacterial species but also fungal strains equally promote plant growth through nutrient recycling, i.e., carbon and nitrogen, provide resistance to pathogens and assist in leaf litter decomposition (Arnold et al., 2007). Various fungal species such as *Penicillium aurantiogriseum*, *Fusarium incarnatum*, *Trichoderma harzianum*, and *Fusarium proliferatum* have been reported from wheat plant (Ripa et al., 2019). Seed-borne endophytic microbes are not fully explored and are of great interest. They potentially produce phytohormones, enzymes, and antimicrobial compounds and improve plant development. The main property of seed endophytes is their vertical transmission. Such microbes are naturally useful in that they signify not only a termination for the community assemblage in the seed, but also an early idea for community gathering in the new seedling (Shahzad et al., 2018). Seed-borne endophytes (bacterial and fungal) benefit seeds by facilitating the germination of seeds in soil.

They are of great interest because they pass their characters to next generation through vertical transmission. This provides important traits in plant growth which are determined by both microbe and plant genomes. Also, seed consists of a limited range of microbial species and has progressed *via* co-selection with the host plant species (Vujanovic et al., 2019). Additionally, this could probably result in reducing the phytopathogenic asset in demand to the sustenance of plant development (Cope-Selby et al., 2017). In addition, they have the ability form endospores and maintain plant growth by phytase activity, regulating cell motility, modulating endogenous phytohormones such as cytokinins that break seed dormancy, enhancing soil structure, and degrading xenobiotics. For instance, fungal endophytes *Epichloe* are stated to support their host plants in growth promotion. Similarly, fungi *Penicillium chrysogenum*, *Trichoderma*, and *Phoma* sp. isolated from *Opuntia* spp. are known to be involved in seed germination (Delgado-Sánchez et al., 2013). In a study, *Paraburkholderia phytofirmans* PsJN actively colonized different seeds of maize, soy, and pepper. Also, wheat seeds colonized with *Paraburkholderia phytofirmans* PsJN showed significant alteration in spike onset compared with non-treated plants under pot and field experiments (Mitter et al., 2017). There are different pathways adapted by seed-borne endophytes. Few enter *via* xylem tissues, through stigma and exogenous pathway where seeds are dirtied from the exterior source. The floral parts of the plant tissue have not been studied extensively for the growth-promoting endophytes. An endophytic fungus, *Lasiodiplodia* sp. ME4-2 isolated from floral parts of *Viscum coloratum* which involved in production of important metabolites regulating plants growth such as indole-3 carboxylic acid and secondary metabolites such as 2-phenylethanol (Qian et al., 2014).

## Endophytic plant growth-promoting mechanisms

**FIGURE 1:**
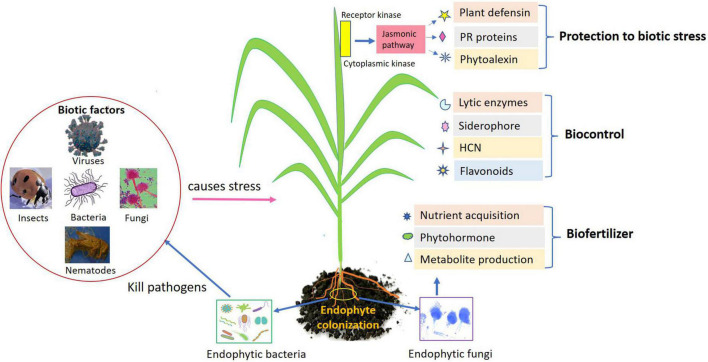
Role of endophytes as biofertilizers, biocontrol, and biotic stress management in agricultural crops.

Endophytes being potential agent impart beneficial effects on their host plant are well-acknowledged inoculants to encourage the plant growth directly/indirectly. Plant growth occurs directly (endophyte–pathogen interaction) through regulating the attainment of vital nutrients such as phosphorous and nitrogen, modulating level of hormones. Indirectly through enhanced plant defense, endophytes could help in biocontrol of phytopathogens by production of antibiotics, regulating defense mechanism by induced systemic resistance, declining the quantity of iron accessible to pathogen, and pathogen inhibition through volatile compounds (Figure 1). Here are the few direct mechanisms involved in plant development.

## Production of phytohormones

Numerous endophytes are identified to produce plant growth hormones (Supplementary Table 1). Hormones stimulate plant growth through regulating structural and morphological changes in response to gravity or light stimuli. They secrete gibberellic acid, cytokinin, auxins such as indole acetic acid, and ethylene. They do not only increase the overall root biomass through enhancing root surface area and root length but are known to act as signal molecules between endomicrobes and plants (Spaepen et al., 2007). In addition, they have been well known to enhance root length and root surface area, control the rate of vegetative growth, and increase the rate at which root and xylem develop. Other indole-related compounds such as indole-3-lactic acid (ILA) and indole acetamide (IAM) also found in different endophytic strains such as *Azospirillum brasilense* which is formed as an intermediate during the auxin biosynthetic pathways. For instance, the root endophyte *Piriformospora indica* produced auxin through utilizing IAA biosynthetic pathway (Xu et al., 2018). The IAA production by endophytes is considered an important factor in plant growth regulation. Khan et al. (2014) reported that *Sphingomonas* sp. (endophyte) isolated from the foliar region of *Tephrosia apollinea* improved growth activity in tomato plants through indole acetic acid (11.23 μm mL^–1^). In another study, *Micrococcus yunnanensis* RWL-2, *Pantoea dispersa* RWL-3, *Micrococcus luteus* RWL-3, and *Staphylococcus epidermidis RWL-7* were analyzed using GC-MS and found to produce IAA (11.50–38.80 μg ml^–1^). When inoculated in rice plants, they significantly increased main growth-promoting attributes in rice plants, i.e., dry biomass, shoot and root length, chlorophyll, and protein contents (Shahzad et al., 2017). Endophytic fungi (*Falciphora oryzae*) helped in lateral root growth while reduced the primary root height (Sun et al., 2020). Also, IAA activity in endophytes also reported to increase nitrogenase activity in rice through showing transcriptional changes in nitrogen-fixing root nodules (Defez et al., 2016). Fungi are also able to produce gibberellins, auxins, and cytokinins important as chemical signaling. Endophytic fungus (*Porostereum spadiceum*) produces gibberellins and rescue growth of soybean under normal and salt affected by promoting seed germination and increasing chlorophyll content (Hamayun et al., 2017). Several endophytic fungi including *A. flavus*, *Paecilomyces formosus*, *P. glomerata*, *Penicillium corylophilum*, *Rhizopus stolonifer*, and *Pochonia chlamydosporia* (Khan et al., 2012). Almost all the gibberellic acid producing fungal endophytes belong to *Ascomycetes* group; however, *P*. *spadiceum* belonging to the Basidiomycota is the first endophyte to produce gibberellic acid and involved in phytostimulation (Waqas et al., 2012). Cytokinins are important group of plant hormones that are involved in apical dominance, chloroplast maturation, cell proliferation and differentiation, seed germination, prevention of senescence, and plant–pathogen signaling mechanisms. Bacterial endophytes *Pseudomonas*, *Sphingomonas*, *Stenotrophomonas*, and *Arthrobacter* sp. isolated from humic-treated cucumber plants produced several cytokinins (cis-zeatin cytokinin, riboside type zeatin, isopentyladenine, and isopentenyladenosine) greater than 30 pmol/ml (De Hita et al., 2020).

## Endophytic diazotrophic bacteria as biofertilizer

Endophytes being successful colonizers of different plants act potentially as biological nitrogen fixers and act as an alternative nitrogen source for crop production. They face less competition over other rhizospheric microbes and directly fix atmospheric N_2_ make it accessible to plants. Moreover, the partial pressure of oxygen inside the plant tissue is suitable in comparison with the outer surface for efficient nitrogen fixation as low partial pressure supports the proper functioning of O_2_-sensitive nitrogenase enzyme (Cocking, 2003). Nitrogen is a vital macronutrient that the plants require because it promotes shoot growth and aid in reproduction and main constituent of chlorophyll. Dinitrogen is an inaccessible form of nitrogen present in air and converted by diazotrophs into soluble, non-toxic form ammonia *via* biological process of nitrogen fixation. The ammonia-oxidizing bacteria and the nitrifying bacteria then transform this ammonia into nitrite and nitrate, respectively. Denitrifying occurs in the deeper soil horizons, converting the unused nitrate to atmospheric nitrogen, which ultimately escapes to the atmosphere as dinitrogen gas. This is the usual nitrogen cycle pathway (Mahanty et al., 2017). Several nitrogen-fixing bacteria have been reported such as *Azospirillum brasilense*, *Acetobacter diazotrophicus*, *Klebsiella oxytoca*, *Rhizobium* sp., and *Burkholderia cepacia* (Kong and Hong, 2020). In addition, various non-leguminous plants such as wheat, sorghum, maize, and rice harbor free-living nitrogen-fixing bacteria. For instance, *Gluconacetobacter diazotrophicus*, *Herbasprillum rubrisubalbicans*, and *Burkholderia silvantantica* can fix nitrogen in the intercellular spaces of sugarcane stems (Lery et al., 2011). Endophytes isolated from rice such as *Bradyrhizobium sp. and Paraburkholderia* sp., showed acetylene reduction properties and high sugar content contributing to high nitrogen-fixing ability. High content of sugar in different crops such as sweet potato, pineapple, and sugar has known to assist endophytic N-fixing activity among non-leguminous plants (Okamoto et al., 2021). *Acetobacter diazotrophicus* and *Azoarcus* isolated from sugarcane and kallar grass potentially fixed atmospheric nitrogen up to 150 kg N ha^–1^ year^–1^ (Gupta et al., 2012).

## Phosphate solubilization

Phosphate solubilization is an important mechanism involved in solubilizing the insoluble phosphate into soluble form like orthophosphate. Plant requires a major amount of phosphorus for enhanced productivity in the range of 30 μmol l^–1^, but limited amount is available to plants which make this nutrient a limiting factor in soil. Endophytes have the capability to solubilize unsolvable phosphates or have the ability to liberate organic phosphates though production of acids such as malic, gluconic, and citric acids. Endophytic bacteria that have been reported to mobilize phosphorus through mineralization and solubilization include *Pseudomonas* spp., *Bacillus megaterium*, *Azotobacter*, *Paenibacillus*, *Thiobacillus*, and *Serratia* (Jahan et al., 2013; Kang et al., 2014).

*Pseudomonas fluorescens* strains isolated from *Miscanthus giganteus* showed great variation in phosphate solubilization capacity with highest solubilization recorded about 1,312 mg L^–1^. Furthermore, when inoculated with the potential strains, high weight of shoot and root was observed in pea plants as compared to control (Otieno et al., 2015). The major endophytic fungi belong to genera *Curvularia*, *Piriformospora*, *Penicillium*, and *Aspergillus* and *Trichoderma.* Symbiotic association of mycorrhizal fungi with plants has been recognized to surge the passage of phosphorus in plants. It is evident from a study that apart from mycorrhizal associations, endophytic bacteria equally contribute to the P solubilization. Poplar samples when inoculated with P solubilizing *Rahnella* and *Burkholderia* sp. strains showed a root architecture with greater root volume under tomography-based root imaging (Varga et al., 2020). Endophytic fungi *Penicillium* and *Aspergillus* isolated form roots of *Taxus wallichiana* solubilized P and produced phosphatase and phytase enzymes (Adhikari and Pandey, 2019). Kang et al. (2014) observed that *Bacillus megaterium* regulates the content of amino acids and carbohydrates to promote the growth of mustard plant.

## Siderophore biosynthesis

Siderophores are low molecular weight composites produced by several microorganisms including endophytes to scavenge iron and make it available to plants. Endophytes are known to synthesize hydroxamate, carboxylate, and phenolate type of siderophore to converse plant protection against phytopathogens. It also assists plant growth and yield by providing iron to plants under iron deficient conditions (Rajkumar et al., 2010). It also facilitates better nutrient mobilization in comparison with rhizospheric counterparts. They are better adapted to the activities of internal tissues of the plants, in terms of originating from the internal microbiome (Verma et al., 2021). Large numbers of bacterial endophytes are there to contain property of iron chelation such as *Azotobact*er, *Bacillus*, *Enterobacter*, *Arthrobacter*, *Nocardia*, and *Streptomyces* (Bokhari et al., 2019).

Biofortification of *Enterococcus hirae* and *Arthrobacter sulfonivorans* in wheat grains not only efficiently makes bioavailability of iron and zinc micronutrients but it also significantly increases plant growth up to 20% in comparison with control (Singh et al., 2018). Bacterial siderophore (catechol and hydroxamate type) isolated from *Arabidopsis thaliana*, *F. rubra* and *Agrostis capillaris*, growing on the heavy metals contaminated area significantly improved growth rate in *Festuca rubra* and *Brassica napus* (Grobelak and Hiller, 2017).

## Role of endophytes as biocontrol agents

Many researchers have previously reported the use of bacterial and fungal endophytes for disease management in plants. *Serendipita indica* conferred resistance against *Fusarium* and *Rhizoctonia solani* and demonstrated antioxidant capacity *in vitro* (del Barrio-Duque et al., 2019). In another study, production of Bacillomycin D protein by *Bacillus amyloliquefaciens* helped in showing antagonistic activity against fungus *Fusarium graminearum* (Gu et al., 2017). Seed application of *B. bassiana* 11-98 efficiently colonized tomato and cotton seedling and protect plants against *Rhizoctonia solani* and *Pythium myriotylum*. Possible mechanisms were coiling of hyphae, induction of resistance, and production of lytic enzymes, thus protecting the older plants from root rot. However, biocontrol practices through endophytes may be achieved through direct inhibition of pathogens or indirectly by establishing the plant’s systemic resistance (Santoyo et al., 2016). The other involved mechanisms include competition for niche and resources, production of cell wall degrading enzymes, initiation of induced systemic resistance (ISR), and quenching the quorum sensing of pathogens (Rajesh and Rai, 2014). Apart from this, several antibiotic compounds and lytic enzymes produced by endophytes reduce disease severity in many plants. For instance, many fungal genera *Fusarium*, *Trichoderma*, and *Botryosphaeria* secrete enzymes such as cellulose, 1,3- glucanases, amylase, and glutaminase which can aid in reducing phytopathogens through inhibiting the cell wall (Ait-Lahsen et al., 2001). Biological control also depends upon many factors such as host specificity, physical structure of soil, inoculum used, and the prevalent environmental conditions. The ability to colonize the plant tissue makes them a better biological control agent than others in having better biological compatibility when applied to plants (Rabiey et al., 2019). Under genomic studies, endophytes were also found to contain several notable genes pertaining to pathogenesis regulation which were previously not found in rhizospheric bioinoculants (Brewer et al., 2016). Also, endophytes are more protected from external factors such as radiations, temperature, and pressure when compared to epiphytes (Andreote et al., 2014). However, a deeper understanding on their mechanism and mode of action is still required to better exploit endophytes as biocontrol agents. Here are the few mechanisms employed by endophytes in controlling diseases in plants.

## Production of secondary metabolites with antifungal and antibacterial properties

Most of the endophytes are known to produce secondary metabolites exhibiting good antibacterial and antifungal activities preventing the growth of harmful microorganisms. Various metabolites such as alkaloids, phenols, flavonoids, peptides, steroids, and terpenoids are isolated from both bacterial and fungal endophytic strains (Supplementary Table 2). Alkaloids possess firm potential in inhibiting the proliferation of microbes. Fungal endophytes such as *Clavicipitaceae* sp. isolated from grass family showed production of alkaloids, which are harmful for aphids (Panaccione et al., 2014). Alkaloids are identified as to contaminate precise hosts and causes slight damage to non-target organisms. Altersetin alkaloid isolated from *Alternaria* spp. displayed a strong antibacterial effect on pathogenic bacteria (Hellwig et al., 2002; Akutse et al., 2013). GS-MS analysis showed production of thermostable metabolites such as d-norandrostane and longifolenaldehyde by *A. alternata* AE1 isolated from neem leaves. Both the compounds have bactericidal and antioxidant properties and showed zone of inhibition against numerous gram-positive and gram-negative bacteria (Chatterjee et al., 2019). Gond et al. (2015) evaluated the effect of antifungal proteins such as iturin A, bacillomycin, and fengycin isolated from *Bacillus* spp. in controlling fungal pathogen *Fusarium moniliforme*. Antifungal protein designated as Efe-AfpA isolated from *Epichloe festucae* showed disease resistance against pathogen *Sclerotinia homoeocarpa* causing dollar spot disease (Tian et al., 2017). Apart from this, many endophytes are widely reported being associated with antibiotic activity. Lipopeptides produced by several endophytes may show antimicrobial and surfactant activities and well known for their antibiotic activity. *Bacillus amyloliquefaciens* strain produces lipopeptides having biocontrol activity toward *Erysiphe cichoracearum* (fungal pathogen). The fengycin, iturin, and surfactin produced by *Bacillus* sp. helped in inhibiting the growth of fungal pathogen. Also, pellicle biofilm formation affected the colonization ability of pathogens (Jiao et al., 2021).

## Bio control strategies through quorum quenching

Quorum sensing (QS) is a signaling mechanism that controls growth and metabolism in single-cell microorganisms such as bacteria. Density-dependent cell-to-cell communication controls most of the traits which are helpful in endophytes as well a key controller of virulence in pathogens (Frederix and Downie, 2011). The factors responsible for virulence such as biofilm formation, toxin production, antibiotic resistance, exopolysaccharides (EPS), and degradative exoenzymes secretions are highly regulated by quorum sensing signaling. This mechanism takes place *via* small diffusible signaling molecules called autoinducers (Seitz and Blokesch, 2013). For instance, many pathogenic bacteria such as *Pseudomonas* and *Ralstonia* primarily use acylated homoserine lactones (AHLs) to communicate while producing virulence (Mansfield et al., 2012). They cause great damage to crops. Therefore, antiquorum sensing approach could be harnessed to trigger the phenotype of pathogen to block infection (Chen et al., 2013). Quenching process is regulated by interfering with virulence-associated activities such as modification of signals, catalysis of degrading enzymes such as AHL-lactonase, and inhibition of signal synthesis (Dong et al., 2002). Lactonase enzyme works through removing the lactone ring from the acyl moiety of AHLs and ultimately inactivates AHLs (Murugayah and Gerth, 2019). Endophytic bacteria and fungi provide plethora of bioactive molecules, which can act as an inhibiting agents including QS quenching enzymes such as lactonase, acyclase, and QS inhibitor molecules (LaSarre and Federle, 2013). Such agents can provide promising approach to control phytopathogens and suppress virulence expression in them. They assist in degrading quorum-sensing signals from pathogenic microbes and disrupt intercellular communication (Rutherford and Bassler, 2012). Endophytes with quorum quenching activity attenuate virulence factors rather than killing the microbes or limit the cell growth. This property effectively reduces the selective pressure associated with bactericidal agents (Chen et al., 2013). QS and *in- silico* analysis showed antiquorum sensing and antibiofilm potential of *Alternaria alternata* isolated from *Carica papaya* against pathogen *Pseudomonas aeruginosa.* Significant decrease in cyanin, alginate, and rhamnolipid production was observed. Protease activity such as LasA protease activity and Las B protease activity responsible for virulence was correlated with decrease in biofilm formation (Mishra et al., 2020).

Endophytes such as *B. firmus* and *Enterobacter asburiae* PT39 showed effective degrading capability of AHL by preventing violacein production (80%) in biosensor strain. Still, cell-free lysate when applied to *P. aeruginosa* PAO1 and PAO1-JP2 biofilm caused decrease in biofilm formation (Rajesh and Rai, 2014). In a study, AHL-degrading bacteria *Pseudomonas nitroreducens* potentially degraded diverse variety of AHL including *N*-(3-oxododecanoyl)-L-homoserine lactone (OdDHL) in *D. Zeac* EC1. It fully degraded OdDHL (0.2 mmol/L) in 48 h. Furthermore, the application of this strain as a biocontrol agent might considerably reduce soft rot disease produced by *D. zeae* EC1 to suppress tissue maceration in numerous host plants (Zhang et al., 2021). These observations demonstrate that QQ strains have huge potential to reduce the disease harshness due to QS-modified pathogenic bacteria. Antivirulence activity can also be achieved by an engineered endophytic bacterium through introducing quorum-quenching gene. For instance, to control *Burkholderia glumae* which causes grain rots of rice, an *N*-acyl-homoserine lactonase (*aiiA*) gene from *Bacillus thuringiensis* was inoculated into *Burkholderia sp.* KJ006 to repress *N*-acyl-homoserine lactone (Cho et al., 2007). Thus, quorum-quenching microbes provide great potential as biocontrol agents. There are several advantages of introducing quorum-quenching microbes into plants. Being compatible in nature endophytes occupies most of the cellular space without leaving space for later-invading phytopathogens (Kung and Almeida, 2014).

## General plant defense responses against biotic stress

Plants are attacked by various pathogens, parasites, and herbivores, all of which cause biotic stress. Various pests belonging to Lepidoptera, Hemiptera, Orthoptera, and Diptera are well known for damage crop plants. Pests destroy more than 40% of the world’s crops every year (FAO, 2021). Also, the fungal parasites are hidden robbers that inhibit the plants growth either by killing the host cell through secretion of toxin or biotropic fungi that feed on living host cell. Host plants become a source of nutrients for such harmful parasites. In some biotropic fungi, haustoria plays a major role in absorbing nutrients from host tissues (Szabo and Bushnell, 2001). Plant viruses also cause leaf chlorosis, spotted wilt, stunted growth in several important plants such as tomato, cucumber, potato, and sugarcane (Roossinck et al., 2015). In addition, nematodes feed on different plant parts (seeds, roots, flowers, leaves, and stems) and cause wounds on the plants. Quick reproduction ability in mites and insects also makes them vectors of other pathogens such as virus and bacteria (Maafi et al., 2013; Adam et al., 2014).

Plants have evolved a plethora of defense mechanisms to combat broad-spectrum pests and pathogens (Rejeb et al., 2014). The defense mechanism could be performed, with toxic metabolites deposited, and it could be inducible. Upon pathogen attack, the innate immune system gets activated that prevents the pathogen entry and terminate their growth. It is a primary defense that contains physical barriers such as waxy cuticles, rigid cell wall, and trichomes to avoid phytopathogens. Cuticle not only restricts the entry of liquid and gas fluxes but also protects plants against pathogens, xenobiotics, and irradiation (Serrano et al., 2014). Trichomes can also have negative or positive effects depending on the target pests through their impact on the behavior of herbivore natural enemies. For instance, the presence of leaf trichomes positively inhabited predatory mite *Typhlodromus pyri* on grapes. On the other hand, European ride mite favored grape varieties with low trichomes (Loughner et al., 2008). Plants can also produce a variety of secondary metabolites to protect themselves from herbivores and harmful microorganisms. Numerous metabolites, such as amines, peptides, alkaloids, cyanogenic glucosides, phenolics, polyacetylenes, non-protein amino acids, and quinines, contribute significantly to disease reduction in plants. Different concentrations and compositions of such compounds work synergistically for defense mechanism (Wink, 2018).

Few defense mechanisms are consecutive (production of phytoanticipins) that are preformed and induced (phytoalexin production) that are activated after pathogen attack. Phytoalexins are low molecular weight compounds that possess antimicrobial. There are wide varieties of phenolic compounds, which assist in phenotypic plasticity and act as inhibitors, pesticides and contain anti herbivory roles (Kant et al., 2015). As rapidly the host plant is infested by pathogen, it displays accretion of phenolics and causes increase in host metabolism. Mainly, hydroquinones, caffeic acid, gallic acids, hydroxycinnamates, and 5-hydroxynapthoquinones are effective allelochemicals (Cheng and Cheng, 2015). Caffeic acid (200 μg/ml) in tobacco root exudates defends tobacco plants from infection by *Ralstonia solanacearum*. It resulted in thinning of cell membrane and created irregular cavities in cells. Moreover, expression of IecM and *epsE* genes associated with inhibition of biofilm formation was also observed and exhibited important prospect in plant defense (Li et al., 2021). In plants, complex network of antioxidative defense system to counter harmful reactive oxygen species (ROS) comprised free radicals such as OH•, O^⋅⁣–^, and non-radicals such as H_2_O_2_ and ^1^O_2_ which are formed under unfavorable circumstances (Huang et al., 2019). ROS scavenging mechanism includes enzymatic components such as catalase, guiacol peroxidase, superoxide dismutase, dehydroascorbate reductase, and glutathione reductase. Non- enzymatic antioxidants such as reduced glutathione, ascorbic acid, carotenoids, and flavonoids help in scavenging oxidative stress (Das and Roy Choudhury, 2014).

**FIGURE 2:**
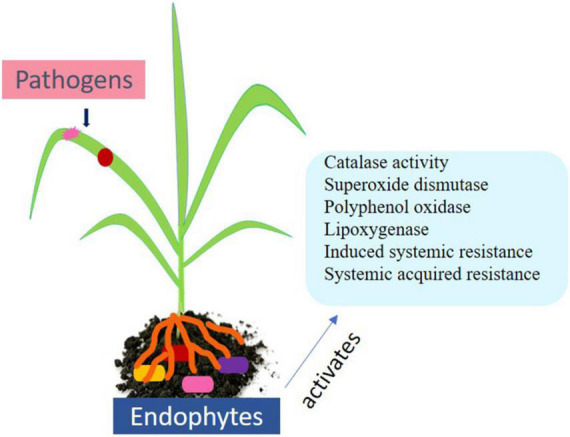
Endophytes and their role in biotic stress management.

Additionally, plant hormones such as salicylic acid, ethylene, and jasmonic acid play central role in biotic stress signaling. Plants also possess an innate immunity system to recognize microbe-associated patterns (PAMP) such as lipopolysaccharides, peptidoglycan, and bacterial flagellin. Such immunity is called PAMP triggered immunity. Herbivores are recognized through herbivore-associated molecular patterns (HAMPs) (Zhang and Zhou, 2010). Other immune response includes transcription methods in the host nucleus and recognizing Avr proteins that are avirulent in nature. Effector triggered immunity arouses hypersensitive responses (HRs) and causes programmed cell death (PCD) in diseased and nearby cells (Howden and Huitema, 2012). A long-lasting and broad-spectrum pathogen resistance against secondary infection known as systemic acquired resistance (SAR) is conserved among diverse plants (Figure 2). Diverse group of molecules including salicylic acid is increased in tissues that occur systematically after localized exposure to a pathogen or after treatment with synthetic or natural compounds (War et al., 2011).

## Endophytes as parasites: Hyperparasitism

It is a biocontrol strategy in which the parasitic host is plant pathogen. In fungi, hyperparasitism is frequently observed, but it is rarely seen in bacteria. Instead of using chemicals, it is frequently used to protect plants against pathogens. *Trichoderma* species, a well-known necrotrophic mycoparasite that targets host mycelium, is the most prevalent hyperparasite (Steyaert et al., 2003; Qualhato et al., 2013). Fungal parasite *Trichoderma harzianum* has a potential ability to parasitize *Epichloe typhina*, an agent that causes choke disease in grasses (Węgrzyn and Górzyńska, 2019). It showed the capability of parasitizing the already-grown mycelium of *E. typhina*. Predatory bacterium such as *Bdellovibrio bacteriovorus* has the uncommon property to use the bacterial cytoplasm as nutrients (Harini et al., 2013). Several pathogenic microbes are predated by *Xanthomonas vesicatoria* including *Erwinia carotovora*, *Pseudomonas syringae*, and *E. herbicola* (McNeely et al., 2017). *Trichoderma* spp. has been found to parasitize *Rhizoctonia solani* hyphae, thus inhibiting the disease production (Harman et al., 2004). This property can be used to treat plant diseases such as damping off in soybean seedlings and root rot in sugar beet.

## Competition for space, infection, and nutrients

Pathogen adapts to nutrient-rich niches such as the rhizosphere, phyllosphere, phloem, and xylem. Pathogens choose different routes into the plant based on their survival needs. Few enter through stomata such as *Pseudomonas syringae*, while others use nectarthodes such as *Erwinia amylovora*, which causes potato fire blight disease (Melotto et al., 2008; Gudesblat et al., 2009). Furthermore, some pathogens have a distinct acquisition strategy and rely entirely on the host plant for nutrition (Fatima and Senthil-Kumar, 2015). Biotrophic pathogens consume nutrients from host tissues. Such pathogens invading plant tissues are competitively prevented by non- pathogenic endophytes already residing in the tissue. Endophytes being ubiquitously present can act through colonization and can resist the pathogen attack through competing for resources which could be available to pathogens through niche overlap. This could be understood from the study by Blumenstein et al. (2015) showing elm (*Ulmus* spp.) endophytes exhibiting extensive niche overlap against Dutch elm disease pathogen. Carbon utilizing profiles of asymptomatic endophytes showed high competition with respect to the utilization of sugar alcohols, monosaccharides, and tri- and tetra-saccharide. In another study, *Lecanicillium* reduced the available nutrients on the leaves while also inducing plant responses during root colonization (Litwin et al., 2020).

## Lytic enzymes as plant disease antagonist

Extracellular enzymes that exhibit biocontrol activity are being increasingly explored as potential antimicrobials to target pathogenic microbes. Numerous endophytes have been reported to produce different lytic enzymes such as chitinase, cellulose, proteases, hemicelluloses, and amylase, which aid the hydrolysis of polymers (Dutta et al., 2014; Bodhankar et al., 2017). Lytic enzymes play vital role in the colonization of endophytes in the host cells through formation of polysaccharide and protein biofilms (Limoli et al., 2015). However, it also helps in controlling plant pathogens through cell wall degradation process (Cao et al., 2009). Specifically, fungal cell wall mostly comprises of polysaccharides that provide structural stiffness to the cell wall in phytopathogens. Therefore, the interference in the glycosidic bonds through enzymatic lysis can deteriorate the cell wall and thereby cause cell death. For instance, extracellular enzyme chitinase isolated from *P. aeruginosa* suppressed phytopathogen *Xanthomonas campestris*, which causes black rot disease in cruciferous vegetables (Mishra and Arora, 2012).

Lytic enzymes chitinases, β 1-3 glucanases, and proteases secreted from *Trichoderma harzianum*, and *Trichoderma viride* significantly reduced the incidence of collar rot disease by *Aspergillus niger* (Gajera and Vakharia, 2012). It assists in the breakdown of glycosidic bond. Similarly, β-1, 3-glucanases synthesized from *Trichoderma harzianum* showed antagonistic activity through hydrolyzing *O*-glycosidic linkage of β- glucan chains in cell wall of parasitic fungi *Sclerotinia sclerotiorum.* It is a serious disease that causes white mold in *Phaseolus vulgaris* (Vázquez-Garcidueñas et al., 1998). However, individual applications of lytic enzymes producers are ineffective, whereas application with another mechanism works well.

## Induced resistance in plants

It is an indirect mechanism through which endophytes inhibit pathogens. Endophytes behold the property to decrease disease susceptibility upon pathogen attack by triggering induced resistance in their host plant (Card et al., 2016). Resistance patterns primarily ISR mediated by phytohormones such as ethylene or jasmonic acid and systemic acquired resistance (SAR) linked with the salicylic acid regulation is the known signaling pathways (Figure 2). Root colonization by endophytes and expression of pathogenesis-related genes is often correlated with the elicitation of induced systemic resistance against infection. For instance, root endophyte *Fusarium solani* has been shown to reduce infection in tomato through activating pathogenesis-related genes such as PR5 and PR7 (Kavroulakis et al., 2007). The endophyte *Bacillus pumilus* along with synthetic benzothiadiazole triggered ISR in contrast to bacterial spot disease in pepper occurred due to *Xanthomonas axonopodis* (Yi et al., 2013). *Fusarium oxysporum* strain Fo47 *via* endophytic-mediated resistance (EMR) was found to suppress various wilt diseases in tomato, flax, watermelon, and pepper (Larkin and Fravel, 1999; Trouvelot et al., 2002). *Epichloe* spp. showed the ability to potentiate expression of salicylic acid defense mechanism against *Blumeria graminis* (Kou et al., 2021). Expression of pathogenesis-related PR1 protein and callose deposition by *Bacillus cereus* induced ISR against *Botrytis cinera* and simultaneously activated the SA- and JA/ET (Nie et al., 2017).

## Modulation of biotic stress controlling mechanisms by endophytes

Microbial endophytes are well identified for their potential role in plant growth-promoting activities. However, their multidimensional interaction with broad range of host plants makes them potential candidate in stress tolerance mechanism (Tamosiune et al., 2017). Endophytic microbes are reported to have numerous beneficial effects in comparison with other PGPRs in colonizing the internal tissues and remain protected from the harsh environment and less nutritional requirement (Pandey et al., 2019). Endophytes commonly reside in plant tissues and benefit their host plant by eliciting defense response toward pathogen outbreak and protect them from different environmental stress (Nanda et al., 2019). Microbial endophytes being inhabitants of plant tissues are known to exhibit unique host’s gene expression, physiological and metabolic response essential in conferring resistance against pests, herbivores, and phytopathogens. Pathogens cause various harmful diseases in plants and interfere with growth mechanisms of plants. It reduces photosynthetic rate, results in stunted growth, and damages plant tissues (Pérez-Bueno et al., 2019). Endophytes produce numerous compounds that help plants to interfere with pathogen by recognizing pathogen related structures. Several metabolites such as volatiles and antibiotics and hormones effectively control the expression genes related to stress response and improve plant growth through induced resistance (Lu et al., 2021).

Some studies reported the similarity of bioactive compounds by endophytic microbes to those formed by host plants (Puri et al., 2006). Different antioxidant enzymes such as peroxidase (POD), polyphenol oxidase, phenylalanine ammonia lyase (PAL), lipoxygenase, and chitinase alleviate biotic stress. Peroxidase enzymes are involved in the wide range of progressions with hypersensitive response, cross-linking of phenolics, lignifications, phytoalexin production, and suberization (Prasannath, 2017). Lipoxygenase belongs to non-heme iron containing deoxygenase that participates in stress response through lipid oxidation and acts as signal molecule to communicate with plants, pathogens, and allied endophytes as reported by Veronico et al. (2006). Different endophytes are known to produce peroxidase enzyme, which play important part in the conversion of H_2_O_2_ into H_2_O as reported by Caverzan et al. (2012). Endophytes boost plant immunity by ISR, SAR, pathogenesis-related proteins and *via* production of numerous phytohormones to overcome the pathogen stress (Romera et al., 2019; Oukala et al., 2021). Several microbes produce surfactin, mycosubtilin, and lipopeptides, which activated the plant innate immune response. It was observed that surfactin production suppresses the *Fusarium* invasion during seed germination (Eid et al., 2021). Suppression of virulence genes such as vir A and vir G and expression of defense-related genes such as PR1, STS, and ANTS induced resistance toward *N. parvum* and *B. cinerea* as reported by Haidar et al. (2016).

## Remodeling and reinforcement of cell wall to cause physical barriers against pathogens

Bacterial and fungal endophytes change chemical and physical characteristic to confer resistance against phytopathogens and herbivory (Supplementary Table 3). High deposition levels of callose in guard cells protect plants from herbivory that cause extensive tissue damage. Callose is β-(1,3)-D-glucan which protects plant tissues from pathogen attack. It is usually deposition among the cell wall and plasma membrane at site of pathogen invasion, at the plasmodesmata and on other plant tissue to slow down pathogen attack (Wang et al., 2021). For instance, endophytic bacteria *B. amyloliquefaciens* and *P. fluorescens* increase callose deposition in guard cells and immunize the *W. somnifera* plant leaves against *A. alternata* (Mishra et al., 2018). Callose deposition and increased lamina density provides resistance to the host plants. It protects plants from different herbivores precisely from leaf wounding ants and aphids (Khare et al., 2018). Upregulation of genes related to cellulose and lignin deposition and hardening of host cell wall were enhanced through inoculation of foliar endophytic fungus *Colletotrichum tropicale* isolated from *T. cocoa.* High cellulose and lignin deposition protects cocoa tree from black pod disease caused due to *Phytophthora* spp. (Mejía et al., 2014). In most cases, fungal endophytes limit insect growth rate, reducing insect survival and oviposition. Consortium of chitinase producing endophytes *Chitiniphilus* sp. MTN22 and *Streptomyces* sp. MTN14 showed uniform lignifications and callose deposition in *B. moneri* protecting against *Meloidogyne incognita* nematode. Callose deposition in leaves was found preferentially in the interveinal region of host leaves (Gupta et al., 2017). Succession of structural variations is observed in *Arabidopsis thaliana* seedlings through callose deposition when inoculated with *Gluconacetobacter diazotrophicus* and protected the plant from *Ralstonia solanacearum* infection (Rodriguez et al., 2019).

Protection efficacy of *B. phytofirmans* PsJN against *Botrytis cinera* was correlated with the callose deposition and H_2_O_2_ production. Further primer expression of PR genes (PR1, PR2, PR5, and JAZ) and modulation in leaf carbohydrate metabolites and sugar levels after pathogen attack were reported from the study (Miotto-Vilanova et al., 2016). Rapid creation of papillae upon pathogen attack especially against fungal pathogens acts as physical fence to limit pathogen entry into the host tissues. Resistance to fungal pathogen is often correlated with the rapid formation of cell wall appositions called papillae, which forms specifically upon interaction between plant and endophytes in response to pathogen attack (Collins et al., 2003). Furthermore, to papillae, phenolic conjugates associated with papillae contribute directly in antifungal activity that forms cross-linking to form a toughened wall that cannot be simply degraded by pathogens and their associated enzymes (Zeyen et al., 2002). These are some successful cell wall-associated defense response mediated through endophytes that can stop invasive pathogens at an initial phase, before the creation of disease in plants.

## Stimulation of bioactive metabolites

Secondary metabolites involved in defense response toward pests, herbivores, and pathogens. Different plant microbes specially endosymbionts secrete various metabolites and regulate defense mechanisms and having antimicrobial properties. Plant secondary metabolites such as steroids, alkaloids, phenolics, flavonoids, and terpenoids function in innate immunity and defense response signaling (Isah, 2019). *Phomopsis* sp. (fungal endophyte) produce VOCs comprised of butanol, acetone, sabinene, 1-butanol, and phenethyl alcohol, which inhibit the *Ascomycetes* and *Deuteromycetes* growth (Singh et al., 2011). VOCs such as caryophyllene, 2-methoxy-4-vinylphenol, and 3,4-dimethoxystyrol having antifungal actions released from *Sarocladium brachiariae* endophytic fungi found to be effective against *Fusarium oxysporum* (Yang et al., 2021). Colonization of asexual *Epichloe festucae* in agricultural forage grasses provided protection against herbivorous insects (Hennessy et al., 2016).

Alkaloid production from Clavicipitaceae and Ascomycota decreases herbivore feeding and virus transmission. Oxidative burst and phytoalexin production improved resistance against *Botrytis cinera* by grapevine cells and leaf-associated bacteria *Pseudomonas fluorescens* (Verhagen et al., 2011). Phytoalexins are low molecular compound containing antimicrobial, antifungal, and antiviral activities, which involved in electron transport and phosphorylation, causes rapid and complete termination of respiration in *B. cinerea* conidia (Pezet and Pont, 1990). Endophytic bacteria (*P. migulae* 8R6) showed ACC deminase activity, which limits the phytoplasma-induced damages in periwinkle through regulating the stress-related hormone such as ethylene. It improved resistance toward infection of phytoplasma and reduced the quantity of symptomatic plants up to 93% (Gamalero et al., 2017). Analysis of free amino acid in diseased leaves showed significant impact of *P. citrinum* and *A. terreus* to disease resistance and promotion of sunflower growth (Waqas et al., 2015). Change in the amino acids delays and changes the progression of pathogenic microbes. Surfactin especially surfactin A and other lipopeptides purified from *Bacillus subtilis*, *Fusarium oxysporum*, *F. moniliforme*, and *F. solani* were known to play major role in antifungal activity (Sarwar et al., 2018).

## Priming of the plant defense system

Endophytes can protect plants against pathogen attack *via* the host by triggering induced resistance *via* several molecular events. Upon pathogen attack, the interaction between plant endophytic associations leads to an alteration in second messenger such as Ca^2+^ in the cytosol (Vadassery and Oelmüller, 2009). It acts as signaling molecule in sensing microbe-associated molecular patterns (MAMPs) and initiates induction of complex immune response. After activation of certain signals, bacterial and fungal endophytes that are attached to cell surface receptors activate kinases (cell surface receptor). When kinases are stimulated, they phosphorylated and send signals to ethylene/jasmonic acid or salicylic acid against phytopathogens which triggers ET/JA transduction pathways (Conn et al., 2008; Ryan et al., 2008). Endophytic colonization with the host plants downregulates the expression of genes associated with biotic stress defense response.

Usually, different phytohormones such as jasmonic acid, ethylene, and salicylic acid triggers induced resistance. JA and ET pathways are known to encourage resistance toward necrotrophic pathogens, but the SA pathway activates resistance toward the biotrophic and hemibiotrophic pathogen (Ding et al., 2011). ISR is normally triggered upon endophytic colonization of roots and immunes the plant body for future attack from pathogens. Several compounds such as flavonoids, polyphenols, phytoalexins, and signal transduction pathways were activated by jasmonate/SA or ethylene (Leon-Reyes et al., 2009; Lebeis et al., 2015). The first report indicating the induced systemic resistance by *Pseudomonas fluorescens* 89B-61 elicited resistance against cucumber anthracnose (Wei et al., 1991). Increased synthesis of phenolic metabolites is often correlated with induced systemic resistance. Contact among *B. distachyon* and *Microdochium bolleyi* (endophytic fungus) isolated from wheat roots induced ISR against pathogen attack of *Fusarium culmorum*. Endophytic fungi upregulated expression of certain genes such as *chitinase 1*, *BdLOX3*, and *TaBH1* induced ISR in wheat (Matušinsky et al., 2022).

Some endophytes can also regulate stress management through SAR mediated by salicylic acid (Pieterse et al., 2014). SA is often associated with building up of pathogenesis-related (PR) proteins and chitinase. *Paenibacillus* strain (PB2) used to control *Mycosphaerella graminicola* induced pathogenesis-related proteins (PR1), which is considered as a marker of SAR (Samain et al., 2019). *Bacillus subtilis* activated a durable defense response in *Arabidopsis thaliana* against *P. syringae* pv. tomato DC3000 facilitated through salicylic acid/ethylene and NPR1 protein (Rudrappa et al., 2010). *Bacillus subtilis* and *Pseudomonas fluorescens*-mediated systemic alleviated the biotic stress in *Solanum lycopersicum* against *Sclerotium rolfsii* (Cappellari et al., 2019). *B. aryabhattai* showed induction of defense-related genes protein (PR1) and phytoalexin-deficient 3 in *A. thaliana*. PR1 gene expression was higher in treated plants (Portieles et al., 2021). Endophytes shows the upregulation of different genes and unique signaling pathway according to dissimilar colonization tactics as reported by Morelli et al. (2020).

There are reports indicating the distinction of endophytic mediated resistance from ISR and SAR as jasmonate, salicylic acid, and ethylene are not involved (Pieterse et al., 2014). Root endophytes *Fusarium oxysporum* strains *Fo 47* and CS-20 have the ability to induce endophytic mediated resistance in tomato and cucumber and protect them against vascular and root pathogens such as *Verticillium dahliae* and *Pythium ultimatum* (Benhamou et al., 2002). Endophytic mediated resistance in case of Fusarium species differs from ISR and SAR in terms of no association of resistance with jasmonic acid and ethylene. Also, tomato plant established a tri-partite interaction with endophytic *Fusarium oxysporum* and other organisms residing in the host plants. Grasses often establish tripartite association among endophytic fungi, arbuscular Mycorrhizal fungi, and *Leymus chinensis* (Liu et al., 2020).

## Defense-related enzymes

Defense mechanisms through endophytes are mediated through the activation of multiple defense compounds and enzymes at the site of pathogen attack. Various enzymes such as PAL, POD, and superoxide dismutase (SOD) are important antioxidant enzymes, which help in defense oxidative stress and lipid peroxidation during pathogen invasion (Birben et al., 2012). Other defense enzymes such as ammonia lyase, chitinase, and β-1-3 glucanase are associated with resistance induction in plants. Several endophytic strains confirmed the production of chitinase enzyme. Some of them are *Colletotrichum sublineolum*, *Streptomyces hygroscopicus*, and *Bacillus cereus*, which are known to inhibit the growth of phytopathogenic fungi such as *Rhizoctonia solani*, *Fusarium oxysporum*, *Aspergillus niger*, and *B. cinerea* (Wang et al., 2001; Brzezinska and Jankiewicz, 2012). ROS that are harmful for plants are neutralized enzymes such as superoxide dismutases, catalases, peroxidase, glutathione-*S*-transferases, and alkyl hydroperoxide reductases. Consortium of *Polyporus vinctus*, *Trichoderma reesei*, and *Sphingobacterium tabacisoli* accumulated defense enzymes such as PAL, POD, and polyphenol oxidase. It triggered systemic resistance contrary to *Fusarium* wilt of banana and showed first line of defense (Savani et al., 2020). Various enzymes are known to mitigate oxidative stress. *Bacillus subtilis* (EPC5) isolated from coconut root samples showed biocontrol activity against *Ganoderma lucidum*, which is the causal agent of basal stem rot on coconut palm through higher induction of phenols, peroxidase, polyphenol oxidase, and phenylalanine lyase (Rajendran et al., 2015).

Evaluation of potential *Streptomyces* spp. viz. *S*. *diastaticus*, *S. olivochromogenes*, *S. collinus*, and *S*. *griseus* triggered systemic resistance and significantly increased total phenolics, flavonoids, superoxide dismutase, ascorbate peroxidase, and guiacol peroxidase which ultimately induced resistance against *Sclertium rolsfii* in chickpea (Singh and Gaur, 2017). Endophytic fungi (*Fusarium sambucinum)* isolated from mangrove forest efficiently produced defense enzymes such as laccase (41.5 U L^–1^), manganese peroxidase (23.6 U L^–1^), endo-xylanase, and biosurfactant (Martinho et al., 2019). These enzymes promote the hydrolysis of lignin and decrease the degree of polymerization exposing the microfibrils to other enzymatic attack. Lipoxygenase genes detected in fungal endophyte *Paraconiothyrium variabile* isolated from conifer *Cephalotaxus harringtonia* showed inhibitory effect on *Fusarium oxysporum*, which causes vascular wilt in conifers. Lipoxygenase genes pvlox1 and pvlox2 unregulated the stress response and acted as stress marker and signaling compound when exposed to invading phytopathogens (Bärenstrauch et al., 2020). It is observed that stress factors affect growth of plants and productivity. In the present situation, thorough and efficient research on the response of endophytes on different essential crops is comparatively inadequate under field conditions. Indeed, understanding the association between crop and beneficial microbes can lead to better agricultural performs that augment plant fitness and improved the yield.

## Molecular mechanism of host–endophyte interaction

It is less understood how the endophyte and host interact. To effectively manipulate the mutualistic link between the two, it is crucial to identify, isolate, and characterize the genes involved in such beneficial interactions. A novel approach for closely examining endophytism and revealing the characteristics required to harbor plants as a habitat has been made available through endophyte genome analysis (Kaul et al., 2016). It has revealed genes important for endophytic lifestyle that are found frequently in endophyte genome such as those involved in N fixation, mineral acquisition, and stress tolerance related (Martinez-Garcia et al., 2015). Exudates such as organic acids, proteins, and amino acids are released by plants from their roots, acting as communications signals between host plant and bacterial endophytes (Kawasaki et al., 2016). Endophytic bacterial colonization is a multistage process that includes chemotactic movement toward roots, attachment to root surfaces, entry inside the root, and movement (Kandel et al., 2017b). There are various genes such as *fliC3*, *MgIB*, *pilX*, *FliI*, *Aer*, and *CheZ*, which involved in chemotaxis and motility (Samanta et al., 2016; Liu et al., 2018). *Gilmaniella* sp. inoculation in *Atractylodes lancea* upregulated the genes and proteins such as terpene skeleton biosynthesis as well as farnesene synthase related to primary metabolism (carbohydrate metabolism, carbon fixation) which improve plant growth (Yuan et al., 2019). Additionally, they noticed an increase in genes related to signaling such as those related to ethylene response factors, heat stress, trielix, and basic loop helices. Sequiterpenoid, phytoalexins such as gossypol and heliocides can protect cotton plants from herbivores infections (Yang et al., 2013). The overexpression of oryzalexin’s genes (*OsTPS19*) and monoterpene S-limonene serve protective metabolite against *Magnaporthe oryzae* and provide resistance to plants toward infection (Chen et al., 2018). Wheat plants have improved resistance to *Fusarium* head blight due to the presence of *Fhb7* gene in endophytic *Epichloe* fungus, which encodes glutathione-S-transferase involved in trichothecenes detoxification (Wang et al., 2020). Dinkins et al. (2017) observed that *Epichloe coenophiala* altered the expression of several WRKY transcription factors linked to the increased resistance in *Lolium arundinaceum*. Endophytic fungus increased the expression of iron transporters and genes involved in fatty acid production to encourage the *Noccaea* plant development (Ważny et al., 2021).

## Omics approach to study endophytes and host plants interaction

Multiomics, which includes genomes, transcriptomics, proteomics, and metabolomics, are becoming increasingly important in plant–microbe interaction (Kaul et al., 2016). The potential value of endophytes can be investigated using modern high-throughput genomic technology. An in-depth examination of endophytes in terms of sequencing and biological evolution has greatly increased interest in endophyte research (Selosse et al., 2022). Endophyte genome-wide analysis directly reflects endophyte colonization preferences and genetic characteristics on various hosts. This makes it much easier to find the related genes involved in host growth, development, insertion elements, metabolism, and surface attachment (Subudhi et al., 2018). *Pantoea ananatis*, an endophytic bacterium with enormous biological potential, contains genes for hydrolase and fusylic acid resistance protein (Wu et al., 2020).

Proteomic analysis using mass spectrometry identified differentially expressed proteins (DEPs) related to the endophytic *Gluconacetobacte*r and sugarcane interaction which involved in signaling and cellular recognition (Lery et al., 2011). Using multiomics analysis, researchers discovered that liposaccharide and adhesins are potential molecular determinants underlying the divergent phenotypic behavior of closely related species during plant–host colonization (Monteiro et al., 2012). RNA sequencing and microarray enables the identification of differentially expressed genes, which involved in upregulation of nutrient acquisition and chemotaxis (*nifH*, *sbpA*, and *trpB*) in wheat roots colonized by *Azospirillum brasilense* (Camilios-Neto et al., 2014). Proteomics and transcriptomics were used to decode the effect of endophytes on the host *Atractylodes lancea* as reported by Yuan et al. (2019). Metabolomic analysis is a popular technique for quantifying metabolites. It can be used to complement transcriptomic and proteomic data, allowing for a well understanding of host phenotypical structures and elucidating plant–microbe interaction and mechanism (Chen et al., 2022). During various stages of plant development, endophytes synthesize a variety of secondary metabolites and mediate an increase in metabolites biosynthesis in particular species and organs (Zhai et al., 2017). The DEGs and metabolites of anthracnose-resistant cultivars of *Camellia oleifera* indicate the critical function of flavonoid biosynthesis in the defense toward anthracnose using transcriptome and metabolomics (Yang et al., 2022). Barley metabolo-transcriptome profiling revealed the activation of the HvCERK1 gene, which confers resistance to *Fusarium graminearum* as reported by Karre et al. (2017).

Microarray-based gene expression analysis revealed single inoculation of endophytic *Bacillus megaterium* isolated from black pepper root encouraged growth elevation in *A. thaliana* Col O seeds by upregulation of biotic stress related genes such as *MYB4*, *MYB7*, *WRR4*, *ATOSM34*, and *ATHCHIB.* Also, the bacterial colonization inside the host tissues triggered ethylene-responsive genes such as *ERF71* and *RAP2.* Other genes *such as BAP1*, *BTK4*, *MKK9*, and *AIBI* were found associated with jasmonic acid and salicylic acid transduction pathways (Vibhuti et al., 2017). In another study, rice seed primed with *Pseudomonas putida* BP25 endogenously colonized rice and altered root growth and defensive response against *Megnaphorthe oryzae.* Defense-related phenols, peroxidase, and both volatile and nonvolatile metabolites were found in primed plants. Also, pathogenesis-related genes associated with systemic acquired resistance, i.e., *OsPR1*-*1* and *OsPR3* were downregulated by endophytic colonization. Growth-related genes playing important role in intermodal elongation such as *OsAcO4* and *OsACS6* were observed regulating plant growth and protecting it against blast disease (Ashajyothi et al., 2020).

Although endophytic microorganisms possess great potential in the agricultural field still, there are certain challenges involved with the field application of endophytes that are restricting their wide use. When introduced into a crop plant, many factors prevail which must be evaluated for their wide application from lab to field. First, many fungal endophytes produce toxic secondary metabolites such as mycotoxins which cause infection in their host plants upon colonization and reach up to fruits and seeds. There is still a need to study upon their colonization and viability of the desired inoculants (Chitnis et al., 2020). It is important to focus on their unpredictable behavior and inadequate colonization of the target site in field trial. Instead of proper establishment of the biological strain, single-strain endophyte inoculants under application do not show desired plant growth activity. Well-formulated consortia could be more promising and help in plant growth promotion through circumventing some of the critical limitations such as crop specificity of microbes. In addition, it is necessary to raise awareness among the farmers about the product’s efficacy of endophytes in comparison with harmful chemical fertilizers. Main attention for the introduction of endophytes is the better understanding of plant–microbe interactions under different sets of conditions that will help in reducing bulk production of inoculum doses (Fadiji and Babalola, 2020). Modifying the root exudation chemistry of plants to choose a more beneficial microbiome is one of the most effective strategies. The use of advance biotechnological tools to investigate both the community and functionalities of endophytic microorganisms could be helpful (White et al., 2019). Understanding the genetics and engineering of their complex interactions through next generation sequencing could be helpful in revealing their taxonomic and functional diversity. However, multiple field trails, sampling at different times and locations under different environmental factors, are an important factor to improve their performance under field conditions. Also, future studies can focus on the development of endophytic nanoparticle which could provide a new aspect of metabolism regulation under extreme condition.

## Conclusion

At present, increasing the productivity of crops is important without any disturbance to the soil fertility, to fulfil food needs and provide a healthy environment for our future generations. But due to the incidence of diverse kind of pest and pathogen in crops, it leads to the decrease in yield of crop plants resulting substantial crop losses every year. To diminish the loss of crop yield and to control the diseases, different effective methods should be used. Endophytes are eco-friendly, non-toxic, easily applicable, and cost-effective in nature, so farmers use them as a substitute to fertilizers for sustainable agriculture. More research needs consideration on the biochemical, molecular, and genetic mechanisms of endophytes decisive for stress resistance in different crops. Omics approach can help unravel the functions of complex plant microbiome, providing information about competent microbes in terms of stress tolerance and plant productivity. Endophytes and their metabolites must be explored to the multiomics level as potentially fruitful research in the biological control of plant diseases.

## Author contributions

PC and AC performed conceptualization and wrote the manuscript. UA wrote the manuscript. AK and GK helped in editing the manuscript. All authors contributed to the article and approved the submitted version.
