# Supplementary material for: Endophytes and their potential in biotic stress management and crop production
Source: Front Microbiol. 2022 Oct 17;13:933017. doi: 10.3389/fmicb.2022.933017 (PMC9618965; doi:10.3389/fmicb.2022.933017)
Supplement: Supplementary file 1 [file Table_1.DOCX]

**Table 1:** **Role of endophytes in plant growth development**

| **Host plant** | **Endophytes** | **Plant growth promotion properties** | **Plant growth parameters** | **References** |
| --- | --- | --- | --- | --- |
| *Ocimum sanctum* | *Bacillus subtilis* | Phosphate solubilization, siderophore production, nitrogen fixation | Involved in enhancement of herb yield | **Tiwari et al., 2010** |
| *Camellia sinensis* | *Stenotrophomonas, Bacillus sp., Alkaligenes sp., Brebibacillus, Pseudomonas, Microbacterium sp.* | IAA, ammonia production and siderophore production, ACC deaminase activity, Hydrolytic enzymes (Protease, amylase, cellulase) | Involved in enhancement of overall growth | **Hazarika et al., 2021** |
| *Teucrium polium* | *Bacillus cereus, Bacillus subtilis, Penicillium chrysogenum, Penicillum crustosum* | IAA and ammonia production, phosphate solubilization | Increased root biomass and plant height | **Hassan, 2017** |
| *Ephedra pachyclada* | *Penicillium*, Alternaria and *Aspergillus sp.* | Produces ammonia and IAA, showed phosphate solubilization and protease activity | Increased biomass | **Khalil et al., 2021** |
| *Stenotrophomonas, Chelatiphaga* | *Pseudomonas*, *Stenotrophomonas*, *Rhizobium*, *Paenibacillus* | Biosynthesis of auxin, siderophore production, phosphate solubilization | Increased root, shoot weight and length in poplar trees | **Ulrich et al., 2021** |
| *Jatropha curcas* L. | *Enterobacte*r R4-368 | Involved in nitrogen fixation | Enhanced seed germination, plant growth and chlorophyll content | **Madhaiyan et al., 2013** |
| *Oryza sativa* | *Microbacterium, Klebsiella, Bacillus, Paenibacillus polymyxa* | Auxin, siderophore and glucanase production, phosphate solubilization | Increase plant growth and dry weight of plants | **Ji et al., 2014** |
| *Phragimates australis* | *P. fluroscens, Pantoea sp., Enterobacter sp.* | Phosphate solubilization and  protease production | Improved seed growth and protect plants from disease | **White et al., 2017** |
| *Phaseolus vulgaris* | *Bacillus thuringiensis, Brevibacillus agri*, *Alternaria sorghi, Penicillium commune* | Ammonia production and phosphate solubilization | Increased plant biomass, photosynthetic pigments, carbohydrate and protein content | **Ismail et al., 2021** |
| *Hordeum brevisubulatum* | *Epichloë bromicola* | Nitrogen and Phosphorus solubilization | Enhanced seed germination, chlorophyll content and metabolic activities of roots under N-deficiency | **Lang et al., 2021** |
| *Populus* | *Enterobacter* sp., *Stenotrophomonas maltophilia*, *Pseudomonas putida* | ACC deaminase activity, siderophore, acetoin and butanediol synthesis | Increase biomass production | **Taghavi et al., 2009** |
| *Tephrosia apollinea* | *Sphingomonas sp. LK11* | IAA production and gibberellins biosynthesis | Increased shoot height and root dry weight in tomato plants | **Khan et al., 2014** |
| *Pulicaria incise* | *Bacillus cereus BI-8*, *Bacillus subtilis* BI-10 | IAA production, phosphate solubilization, ammonia production | Enhanced plant height, fresh weight of root and shoots, dry weight of root and shoot | **Fouda et al., 2021** |
| *Glycine max* | *Bacillus cereus, Pseudomonas otitidis* | Phosphate solubilization, IAA, HCN and ammonia production and showed ACC deaminase and catalase activity | Improvement in biomass, stem length and root length | **Dubey et al., 2021** |
| *Musaceae* | *Enterobacter cloacae* | N- fixation, ACC deaminase, P solubilization | Increased weight biomass (root and shoot fresh weight) and plant height | **Macedo- Raygoza et al., 2019** |
