# Supplementary material for: Endophytes and their potential in biotic stress management and crop production
Source: Front Microbiol. 2022 Oct 17;13:933017. doi: 10.3389/fmicb.2022.933017 (PMC9618965; doi:10.3389/fmicb.2022.933017)
Supplement: Supplementary file 2 [file Table_2.DOCX]

**Table 2:** Secondary metabolites obtained from bacterial and fungal endophytes and their pharmacological importance

| **Endophytes** | **Host plant** | **Secondary metabolites** | **Biological activity** | **References** |
| --- | --- | --- | --- | --- |
| Arthrinium sp. | Zingiber cassumunar | β-cyclocitral, 3E-cembrene A, laurenan-2-one, sclareol, 2Z,6E-farnesol | Antibacterial and antioxidant | **Pansanit and Pripdeevech, 2018** |
| *Nigrospora sphaerica* | *Vinca rosea* | Ergosta | Antifungal activity | **Metwaly et al., 2014** |
| *Fusarium oxysporum* | *Sceletium tortuosum* L. | 5-hydroxymethylfurfural, octadecanoic acid | Antibacterial activity | **Manganyi et al., 2019** |
| *Curvularia*  *Tsudae* | *Cynodon dactylon (L.)* | Esculetin, 4-hydroxycoumarin, isofraxidin and scopoletin | Antimicrobial, antioxidant properties | **Nischitha et al., 2020** |
| Aspergillus fumigatus | Cordyceps sinensis | 3,6-dihydroxy-8-methoxy-3-methylisochroman | Anticancer activity | **Li et al., 2019** |
| *Colletotrichum gloeosporioides* | *Piper nigrum* | Piperine | Antimicrobial, antidepressant, anti- inflammatory | **Clithra et al., 2014** |
| *Cochliobolus nisikadoi* | *Cinnamomum* | Borneol | Anti- inflammatory, antioxidant | **Chen M. et al., 2011** |
| *Rhizopus oryzae* | *Iris germanica* | α – Irone and β- Irone | Anti- inflammatory | **Lingqi et al., 1999** |
| *Sordariomycete* sp*.* | *Eucommia ulmoides* | Chlorogenic acid | Antimicrobial and antitumor | **Chen et al., 2010** |
| *Bacillus atrophaeus* | *Glycyrrhiza* | 1,2 - benzenedicarboxylic acid, 9,12- octadecadienoic acid | Antifungal activity | **Mohamad et al., 2018** |
| *Enterobacter* | *Thymus vulgaris* | Acetic acid, butyl ester; benzene, 1.3- dimethyl-propane,1- ethoxy | Antifungal and antioxidant property | **Abdelshafy Mohamad et al., 2020** |
| *Streptomyces* | *Vochysia divergens* | Indole-3-carbaldehyde, 1-acetyl-β-carboline | Antifungal activity | **Gos et al., 2017** |
| *Lysinibacillus* | *Alectra sessiliflora* | 4-mecrcaptophenol, 2, 4-di- tertbutyl phenol | Antibacterial and antitumor property | **Maela et al., 2022** |
| *Bacillus velezensis* | *Arachis hypogaea* | Fengycin and surfactin | Antimicrobial | **Chen et al., 2019** |
| *Micromonospora echinospora* | *Ibervillea sonorae* | Methyl and ethyl acetate extract | Antitumor ctivity | **Romero-Arguelles et al., 2022** |
| *Pseudomonas aeruginosa* | *Anredera cordifolia* | Diisooctyl phthalate,  oxadiazole, 5-benzyl-3 | Antibacterial and antioxidant activities | **Nxumalo et al., 2020** |
