# Supplementary material for: Endophytes and their potential in biotic stress management and crop production
Source: Front Microbiol. 2022 Oct 17;13:933017. doi: 10.3389/fmicb.2022.933017 (PMC9618965; doi:10.3389/fmicb.2022.933017)
Supplement: Supplementary file 3 [file Table_3.DOCX]

**Table 3:** Role of bacterial and fungal endophytes in biotic stress tolerance

| **Endophytes** | **Host plant** | **Pathogen** | **Response** | **References** |
| --- | --- | --- | --- | --- |
| *Bacillus subtilis* | *Atractylodes macrocephala* | *Ceratobasidium* sp. | Inhibit the mycelial growth of pathogen and promote plant growth | **You et al., 2018** |
| *Bacillus subtilis* | Coconut palm | *Ganoderma lucidum* | Increased production of phenols and peroxidase | **Rajendran et al., 2015** |
| *Bacillus amyloliquefaciens* | *Lichi chinensis* | *Peronophthora litchi* | Upregulation of pathogen related proteins and chitinases | **Cai et al., 2010** |
| *Bacillus amyloliquefaciens* | *Pear* | *Botryosphaeria berengeriana* | Inhibit pathogen growth and induce the production of POD and CAT | **Pingping et al., 2017** |
| *Bacillus cereus* | *Arabidopsis* | *Botrytis cinerea* | Regulates signalling pathway such as JA and MAPK | **Nie et al., 2017** |
| *Bacillus velezensis* | *Arabidopsis* | *Myzus persicae* | Protects host from pathogen via systemic resistance response | **Rashid et al., 2017** |
| *Gluconacetobacter diazotrophicus* | *Arabidopsis thaliana* | *Ralstonia solanacearum* | Protects from pathogen and activates defense response in plants | **Rodriguez et al., 2019** |
| *Gluconacetobacter diazotrophicus* | *Sugarcane* | *Xanthomonas albilineans* | Increased production of bacteriocins which inhibit the pathogens | **Blanco et al., 2005** |
| *Pseudomonas aeruginosa* | Cruciferous vegetables | *Xanthomonas campestris* | Chitinase production which protect plant from disease | **Mishra and Arora, 2012** |
| *Pseudomonas fluorescens* | *Withnia somnifera* | *Alternaria alternata* | Improved plant growth | **Mishra et al., 2018** |
| *Aureobasidium pullulans, Sarocladium pullans* | Olive trees | *Colletotrichum acutatum* | Increased production of volatile fatty acids and improve germination | **Sdiri et al., 2022** |
| *Cladophialophora chaetospira* | *Strawberry* | *Fusarium oxysporum* | Improved flower formation and photosynthetic rate | **Harsonowati et al., 2020** |
| *Epichloe festucae* | *Festuca rubra* | *Sclerotinia homeocarpa* | Increased secretion of antifungal proteins and protects plant from disease | **Tian et al., 2017** |
| *Epichloe festucae* | *Lolium perenne* | *Bipolaris sorokiniana* | Improved plant growth and photosynthetic parameters and P uptake | **Liu et al., 2020** |
| *Epichloe gansuensis* | *Achnatherum inebrians* | *Blumeria graminis* | Decrease colonization of pathogen and improved plant growth | **Xia et al., 2015** |
| *Funneliformis mosseae* | *Solanum lycopersicum* | *Alternaria solani* | Increased chitinase and glucanase production | **Song et al., 2015** |
| *Harpophora oryzae* | Rice | *Magnaporthe oryzae* | Production of ROS and protects plants from pathogens | **Su et al., 2013** |
| *Phialocephala fortinii* | *Asparagus*  *Officinalis* | *Fusarium oxysporum* | Alleviation the disease in plants | **Surono and Narisawa, 2018** |
| *Phialocephala bamuru* | *Pinus sylvestris* | *Rhizoctonia solani* | Increased production of proline and chitinase activity | **Deng et al., 2020** |
| *Trichoderma harzianum* | *Millet* | *Colletotrichum acutatum* | Increased IAA production and improved plant growth | **Saber et al., 2017** |
